# Clinical efficacy of fecal microbial transplantation treatment in adults with moderate‐to‐severe atopic dermatitis

**DOI:** 10.1002/iid3.570

**Published:** 2021-12-20

**Authors:** Jacob Mashiah, Tal Karady, Naomi Fliss‐Isakov, Eli Sprecher, Dan Slodownik, Ofir Artzi, Liat Samuelov, Eran Ellenbogen, Anastasia Godneva, Eran Segal, Nitsan Maharshak

**Affiliations:** ^1^ Division of Dermatology and Venereology Tel‐Aviv Sourasky Medical Center Tel‐Aviv Israel; ^2^ Pediatric Dermatology Unit, Dana Children's Hospital Tel‐Aviv Sourasky Medical Center Tel‐Aviv Israel; ^3^ Sackler Faculty of Medicine Tel‐Aviv University Tel‐Aviv Israel; ^4^ Department of Computer Science and Applied Mathematics Weizamnn Institute of Science Rehovot Israel; ^5^ Department of Gastroenterology and Liver Diseases Tel Aviv Medical Center Tel Aviv Israel

**Keywords:** atopic dermatitis, fecal microbial transplantation

## Abstract

**Background:**

Atopic dermatitis (AD) is a remitting relapsing chronic eczematous pruritic disease. Several studies suggest that gut microbiota may influence AD by immune system regulation.

**Methods:**

We performed the first in‐human efficacy and safety assessment of fecal microbiota transplantation (FMT) for AD adult patients. All patients received 2 placebo transplantations followed by 4 FMTs each 2 weeks apart. AD severity and fecal microbiome profile were evaluated by the Scoring Atopic Dermatitis Score (SCORAD), the weekly frequency of topical corticosteroids usage, and gut microbiota metagenomic analysis, at the study beginning, before every FMT, and 1–8 months after the last FMT.

**Results:**

Nine patients completed the study protocol. There was no significant change in the SCORAD score following the two placebo transplants. The average SCORAD score significantly decreased from baseline at Weeks 4–12 (before and 2 weeks after 4 times of FMT) (59.2 ± 34.9%, Wilcoxon *p* = .011), 50% and 75% decrease was achieved by 7 (77%) and 4 (44%) patients, respectively. At Week 18 (8 weeks after the last FMT) the average SCORAD score decreased from baseline at Week 4 (85.5 ± 8.4%, Wilcoxon *p* = .018), 50% and 75% decrease was achieved by 7 (77%) and 6 (66.7%) patients respectively. Weekly topical corticosteroids usage was diminished during the study and follow‐up period as well. Two patients had a quick relapse and were switched to a different treatment. Two patients developed exacerbations alleviated after an additional fifth FMT.

Metagenomic analysis of the fecal microbiota of patients and donors showed bacterial strains transmission from donors to patients. No adverse events were recorded during the study and follow‐up period.

**Conclusions:**

FMT may be a safe and effective therapeutic intervention for AD patients, associated with transfer of specific microbial species from the donors to the patients. Further studies are required to reconfirm these results.

## Graphical abstract

Fecal microbiota transplantation may be a safe and effective therapeutic intervention for AD patients, associated with transfer of specific microbial species from the donors to the patients.

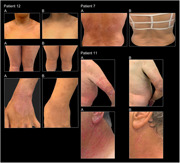

## 1 INTRODUCTION

Atopic dermatitis (AD), a remitting‐relapsing chronic eczematous pruritic skin disease, affects up to 20% of children and up to 10% of adults.^1^ The etiology of AD is multifactorial comprising of genetic predisposition, immune dysregulation, and immune deviation mainly towards T_H_2/T_H_22 with some T_H_1 and T_H_17 overexpression, defective skin barrier function, abnormal microbial colonization, as well as environmental factors. Nearly 20% of all cases are considered as moderate or severe, causing quality of life impairment, psychological, social, as well as financial burdens.^2^

Until recently, the therapeutic ladder of AD consisted of topical treatments, phototherapy, and immunosuppressant agents.^3^ Dupilumab, a fully human, monoclonal antibody inhibiting signaling of both interleukin (IL)‐4 and IL‐13 is the first and only targeted biologic treatment approved for moderate to severe AD until now.^2^

Several studies addressed the role of microbiota in AD, with much of the emphasis being put on the skin microbiota, in particular Staphylococcus aureus.^4^, ^5^ However, recent evidence supports the importance of the gut‐skin axis, probably through the immune regulatory and immune activation capabilities of gut microbial antigens and metabolites.^6^ According to the hygiene hypothesis there is an inverse relationship between AD and an early exposure to microbial agents.^6^ Gastroenteritis during infancy as well as exposure to antibiotics during the first year of life are associated with AD in children.^7^, ^8^ Indeed, gut microbial dysbiosis has been demonstrated in AD patients.^9^ Moreover, in some studies, probiotics were found to have a positive effect on AD severity, alter the gut microbiota, and possibly propagate induction of regulatory T cells. A recent study using mouse model of AD showed that fecal microbiota transplantation (FMT) was associated with restoration of gut microbiota and immunological balance (Th1/Th2) along with suppression allergic response.^10^, ^11^

In this study, we evaluated the effect of fecal microbiome transplantation in AD patients.

## 2 METHODS

### 2.1 Trial population

AD patients treated at the Dermatologic department of the Tel Aviv Medical Center, who were ≥18 years of age, with moderate‐to‐severe AD, as defined by a Scoring Atopic Dermatitis (SCORAD) score ≥25, with minimum disease duration of 3 years, inadequately controlled by topical and systemic therapy. Key exclusion criteria comprised of another concomitant active dermatologic disease, pregnancy, and systemic therapy including antibiotics and phototherapy within 4 weeks before the beginning of the study.

### 2.2 Study design and oversight

**Figure 1:**
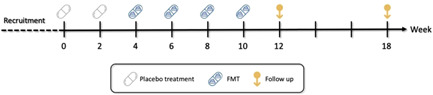
Study design: Placebo and FMT schedule. FMT, fecal microbiota transplantation

This was a proof of concept, single‐blinded, placebo‐controlled cross‐over pilot study addressed to assess the safety and efficacy of FMT for the treatment of mild‐moderate AD, and to assess the change in the fecal microbiota following FMT. All patients received 2 doses of placebo FMTs, followed by 4 treatment FMTs (from healthy stool donors) each 2 weeks apart (Figure 1). Each patient received FMTs from a single donor (one of 3 available stool donors). However, if stool donations from a certain donor were no longer available, stool donor was changed. The clinical activity of AD, adverse events and the fecal microbiome profile were evaluated at the beginning of the study, before every FMT, and 1–8 months after the last FMT, using the SCORAD score, the weekly usage of topical corticosteroids, and gut microbiota metagenomic analysis.

During the study period, patients could use only topical therapy including emollients and glucocorticoids or calcineurin inhibitors.

The protocol was approved by the ethics review board of the Tel Aviv Medical Center. All patients provided written informed consent in accordance with the principles of the Declaration of Helsinki. There was no commercial support for the trial. All the authors vouch for the accuracy and completeness of the data, for the adherence of the trial to the protocol, and for the reporting of adverse events.

### 2.3 FMT preparation and delivery

The three volunteer stool donors were healthy, nonpregnant adults aged 18–50 years, with a normal body mass index of 18.5–24.9 kg/m^2^, excluded for any significant medical history or for any use of antibiotics in the preceding 3 months. They were eligible according to the Israeli Ministry of Health guidelines which include a physical examination and elaborative laboratory screening tests, including fecal enteric pathogens (through cultures, and PAN‐PCR testing [Biofire Diagnostics, gastrointestinal panel]), serum antibodies to hepatitis A, B, and C; human immunodeficiency virus; HTLV, and Treponema pallidum as well as celiac, CBC (and additional tests that comply with the guidelines of the Israeli Ministry of Health). Preparation of capsulized FMT was previously described.^12^ Placebo capsules were identical visually and contained diluted glycerol only.

### 2.4 Capsulized FMT procedure

Two doses of 15 FMT capsules were administered on 2 consecutive days. Patients were asked to fast overnight before capsule intake.

### 2.5 Efficacy end points

The study primary endpoint was the overall mean change from baseline of the SCORAD score.^13^, ^14^ The secondary endpoint was safety of FMT for AD patients and the relation of clinical improvement to the change in the fecal microbial species 2 weeks after each FMT, and 8 weeks or more after the last FMT.

### 2.6 Fecal microbial analysis

Stool samples collected from the donors, and the patients during the study period were sequenced into metagenomics reads using Illumina NextSeq. We used bowtie2^15^ to map reads to reference genomes dataset which is based on the representative assembly of the species‐level genome bins (SGBs) defined by Pasolli et al.^16^ The reads were piled up to obtain per‐position variant information for every detected species. Difference in the variant of a particular species at a given position between two samples was defined as having no intersection between the set of detected alleles in the two samples being compared. The estimated species DNA sequence dissimilarity for a pair of samples is then the number of different positions divided by the total number of positions being compared (CP):

$$\text{Dissimilarity}(S,x,y,d)=\frac{1}{\text{CP}}d\left((,x\left[[,i,]\right],y\left[[,i,]\right],)\right)$$

for species S, samples x, y with minimum of 20 kbp CP, and a comparison method d.

The CP cut‐off means that dissimilarities below 0.00005 (=1/20,000) changes per base pair can differ due to a different number of CP. We therefore define the minimal dissimilarity detection threshold to be 0.00005 changes per base pair.

Dissimilarities below the defined detection threshold are referred as “transmission events” or “strain sharing”. An aggregated dissimilarity score was defined for pair of samples as:

$${\text{sample}}_{\text{dissimilarity}}\left((,X,Y,)\right)=1-\frac{N_{s}}{1+N_{c}}$$

where $N_{S}$ is the number of shared strains between samples X and Y and $N_{C}$ is the number of comparable species between samples X and Y.

### 2.7 Statistical analysis

Statistical analysis was performed with Python scipy.stats package. The Wilcoxon signed‐rank test for comparison between two matched samples was used for calculation of significance levels between different groups. Correlations were calculated according to Pearson correlation method.

## 3 RESULTS

A total of 15 AD patients were enrolled to the trial. Five did not enter the study due to inability to comply with the trial regimen. Another patient (patient 6) breached the study protocol by using enemas, thus, possibly effecting the gut microbiome composition. Nine patients (5 males and 4 females), with average age of 44.6 years (range: 24–68 years), completed the study. One of these patients (patient 8) failed to deliver stool samples and is, therefore, not included in the fecal microbiome analysis.

Before treatment, the average SCORAD score of all 9 patients was 51.2 ± 16.2, with 4 patients classified as moderate (SCORAD 25–50), and 5 as severe (SCORAD >50). The average usage of topical corticosteroids was 10.4 ± 3.5 applications per week. Before enrolment all patients received topical therapy as well as one or more systemic treatment (Table 1).

**Table 1:** Patients' characteristics and SCORAD scores during the study period

Patient No.	2	3	7	8	10	11	12	13	15
Characteristic									
Age (years)	41	56	68	45	35	59	24	37	37
Sex	F	M	F	F	M	M	F	M	M
Previous Tx	PT	PT	PT, CS	PT, CsA	CS	PT, MTX, IL‐4i	CS	CsA, CS	PT, CS
Treatment (SCORAD score)									
W0—Placebo	70	41	70	31	27	41	61	57	61
W4—FMT1	67	39	48	32	27	62	71	60	55
W6—FMT2	41	18	4		14	29	15	28	49
W8—FMT3	25	12	4	7	9	17	22	44	38
W10—FMT4	33	10	4	14	11	19	29	22	39
W12	30	6	0	40	11	10	30	11	34
W18	11	5	0		4	8		10	14
Last follow‐up (W)	10 (W29)	5 (W32)	5 (W33)	40 (W12)	5 (W23)	8 (W18)	30 (W12)	11 (W18)	14 (W18)
Extension—FMT5 (W)	49 (W42)					36 (W31)			
Extension—follow‐up (W)	10 (W46)					22 (W34)			

*Note*: W—week, Tx—treatment, PT—phototherapy, CS—systemic corticosteroids, CsA—Cyclosporine, MTX—Methotrexate, IL‐4i— Dupilumab.

Abbreviation: SCORAD, Scoring Atopic Dermatitis.

**Figure 2:**
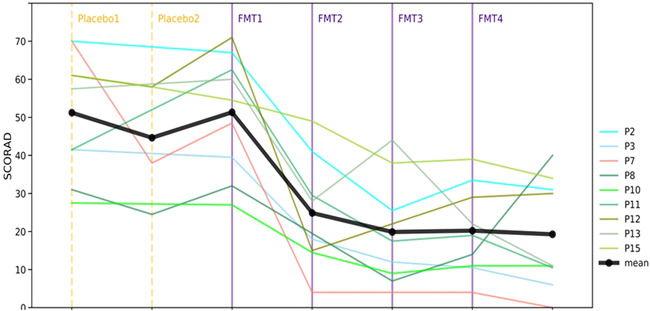
Changes in patients' SCORAD measurements across study time according to placebo treatments and FMTs. Black line represents the average SCORAD of eligible patients. Vertical line indicates placebo treatments and FMTs. FMT, fecal microbiota transplantation; SCORAD, Scoring Atopic Dermatitis

**Figure 3:**
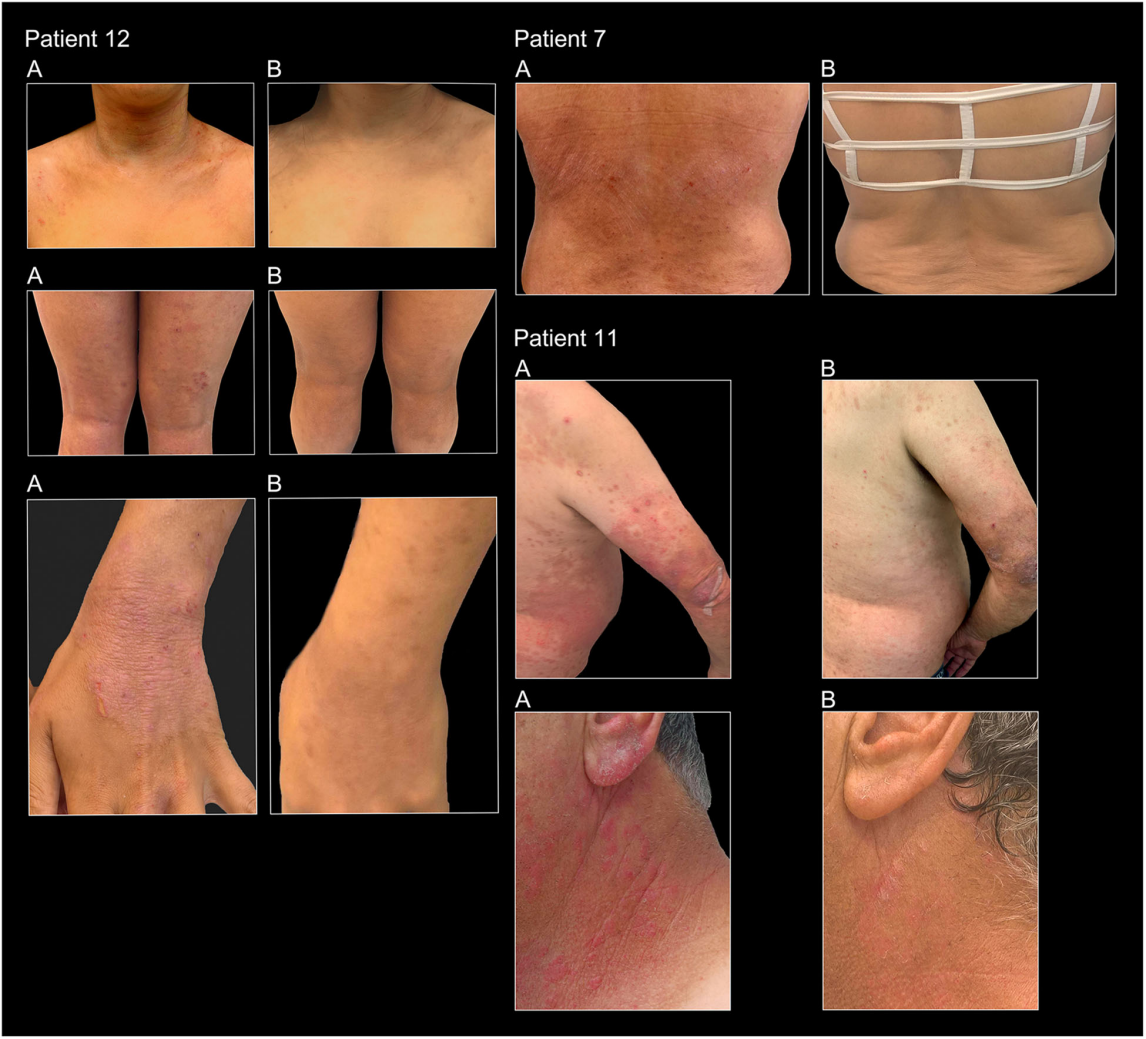
Pictures of patients 7, 11, 12. All A: Pictures taken at Week 4 (after 2 placebo transplantations and before the FMT treatment). All B: Pictures taken at Week 12 (2 weeks after 4 sessions of FMT). FMT, fecal microbiota transplantation

After the placebo part, there was no significant change in AD severity (SCORAD score augmentation of 2.5 ± 22.1%, Wilcoxon *p *= .859), and the number of weekly applications of topical corticosteroids (reduction of 6.3 ± 19.0%, Wilcoxon *p* = .317). One patient exhibited improvement of the SCORAD score of 31%, four patients exhibited improvement in the range of 1%–11%, and the remaining four patients experienced worsening by an average SCORAD elevation of 19% (range: 3%–51%). All 9 patients who started the study completed the FMTs protocol. Following each FMT there was a significant reduction in the SCORAD score compared to the score at Week 4, (after 2 placebo transplantations and before the FMT treatment) (53.3 ± 23.1%, Wilcoxon *p* = .012, 62.9 ± 20%, Wilcoxon *p* = .008, 61.2 ± 16.3%, Wilcoxon *p* = .008, and 59.2 ± 34.9%, Wilcoxon *p* = .011, respectively) (Figures 2 and 3), as well as significant reduction in the number of weekly topical corticosteroids applications (43.0 ± 41.4%, Wilcoxon *p* = .043, 58.3 ± 27.5%, Wilcoxon *p *= .012, 59.4 ± 17.7%, Wilcoxon *p* = .007, 56.8 ± 43.0% Wilcoxon *p* = .010, respectively). At Week 12 (2 weeks after the last FMT), 50% and 75% decrease of the score, compared to the score before FMT (Week 4) was achieved by 7 (77%) and 4 (44%) out of the 9 patients, respectively. Two participants had a quick relapse and were switched to a different treatment. Seven patients continued follow up and at Week 18 (8 weeks after the last FMT) the average SCORAD score continued to decrease (85.5 ± 8.4%, Wilcoxon *p* = .018) and 50% and 75% SCORAD decrease was achieved by 7 (77%) and 6 (66.7%), respectively. The frequency of the weekly topical corticosteroid usage was diminished during the follow‐up period as well (90.5 ± 10.7%, Wilcoxon *p *= .008).

**Figure 4:**
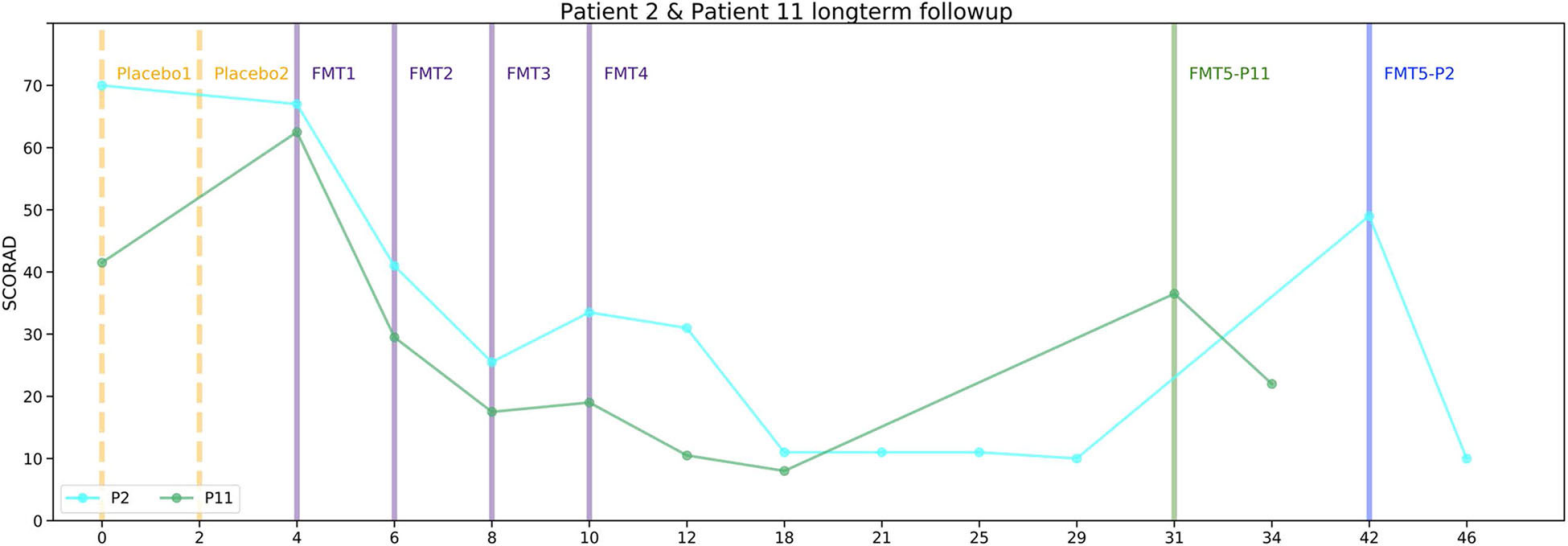
Measurements change in long‐term follow‐up patients. Patients 2 and 11 SCORAD across time by weeks from the beginning of the study. Vertical lines indicate placebo treatments and FMTs. FMT, fecal microbiota transplantation; SCORAD, Scoring Atopic Dermatitis

Two patients (No 2 and 11) had an exacerbation, reflected by an increase of the SCORAD score from 11 to 49, and from 8 to 36, 32 and 21 weeks after the last FMT, respectively, still a reduction from their baseline score. Additional single FMT led to SCORAD score decrease of 80% and 40% respectively (Figure 4).

Most patients received all FMTs from one donor, however, three patients received FMTs from two donors, without significant response rate difference. No adverse events were reported during the study.

### 3.1 Transmission of bacterial strains from donor to patient

When comparing patient‐donor dissimilarities across different study time points to both inter‐host and intra‐host dissimilarities in a healthy reference population of more than 1200 subjects,^17^ we found many (691 of 2052 comparisons, 33.7%) cases of low dissimilarity between patients and their donors after the FMT, but rarely comparing inter‐host dissimilarities in our healthy population and also when comparing patients to their donors at either baseline (0 of 244 comparisons, 0%) or after two doses of placebo (0 of 277 comparisons, 0%).

Overall, we detected 114 transmission events from donors to patients, with an average of 14.3 ± 8.3 transmission events per patient, and at least two transmission events were found during the intervention period. Overall, fifty different species were transmitted, of which 8 species were from the Prevoellaceaca family and 9 species from the Lachnospiraceae family. Thirty‐one species were transmitted into more than one patient, with Prevotella copri (SGB_1626) standing out as it was transmitted in 7 of the 8 patients.

For most patients, we found high similarity between strains at baseline and after placebo treatment, indicative of high microbiome stability. In contrast, following the FMTs, we found high dissimilarities to strains at baseline, indicative of transmission events and changes in the microbiome strain pool. Comparing patients to their donors, we found a mirror picture, whereby there was high dissimilarity between the donor and both baseline and postplacebo samples of each patient, and low dissimilarity after the FMT. Together, these results suggest that without any treatment (including placebo) bacterial strains of patients are stable over time but following FMT some strains are transmitted from donors and replace previous strain that exist in patients.

### 3.2 Patients' gut microbiota becomes similar to the donor's following FMT

**Figure 5:**
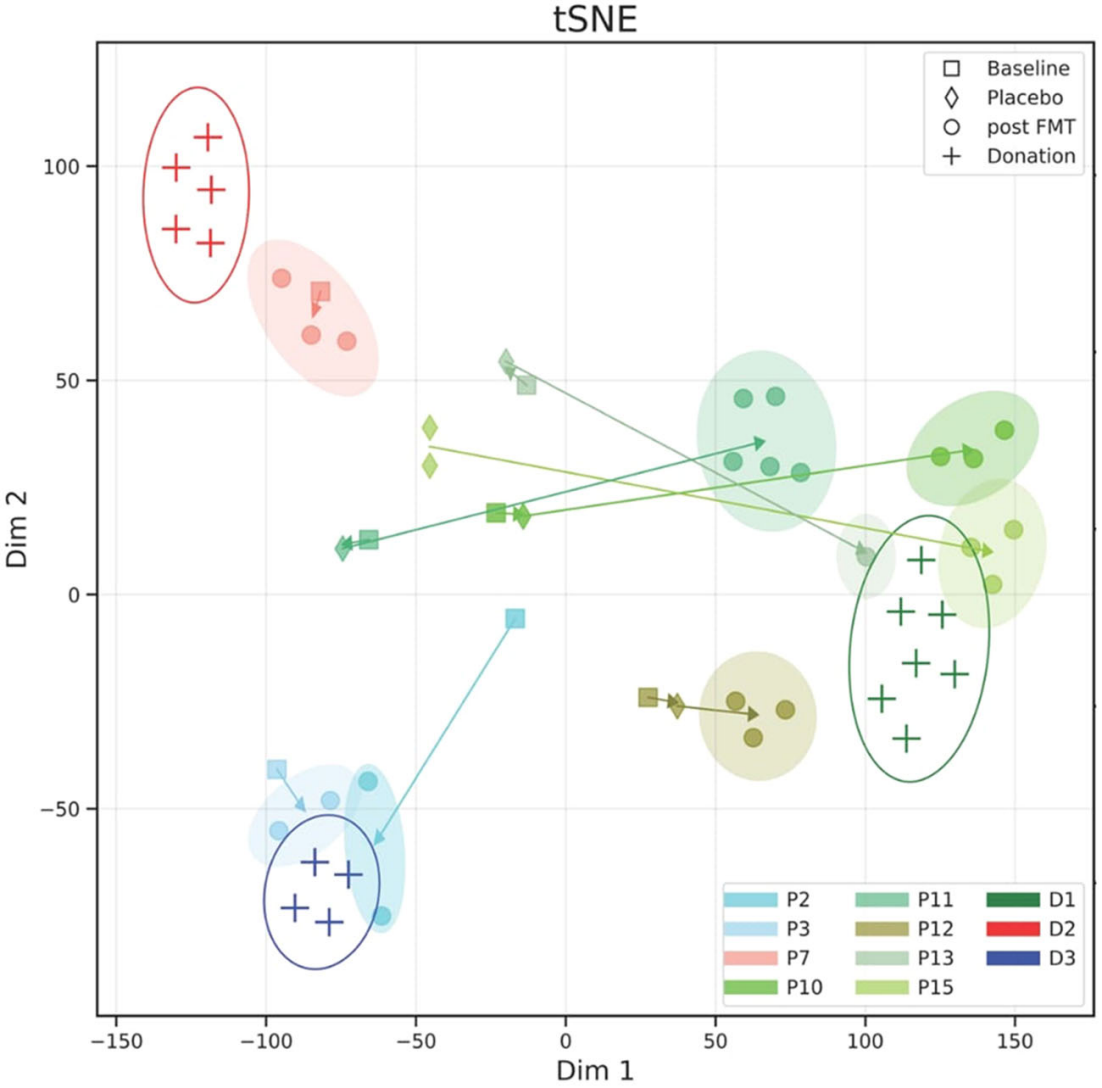
Patient samples become similar to donor samples following FMT. t‐SNE (t‐distributed stochastic neighbor embedding) analysis based on sample dissimilarity matrix of patients with available pre‐FMT and post‐FMT samples (*N* = 8) and donors' samples (*N* = 3), showing shift of patient samples towards their donor's cluster. Arrows demonstrate the path from baseline samples (square markers), through placebo samples (diamond markers) to post‐FMT samples (circle markers). Color scale matches the patient's main donor. Empty ellipses encompass the donors' samples and filled ellipses encompass patient post‐FMT samples. FMT, fecal microbiota transplantation

We performed a t‐distributed stochastic neighbor embedding analysis on the pairwise dissimilarity matrix of all samples (Figure 5). This global view demonstrates that baseline and placebo samples are randomly distributed across the space while post‐FMT samples of patients' cluster near their donor samples. Thus, following FMT treatment, strains in the microbiome composition of patients becomes similar to those of their donors. A similar analysis was performed on a Bray‐Curtis dissimilarity matrix that was computed based on the relative abundance data of the samples. In this case, most of the patients' post‐FMT clusters are randomly distributed across the space and are not located near their donor, suggesting that the effect of the FMTs is more reflected by specific strains transmission than by changes in the relative abundance.

### 3.3 Patient to donor fecal sample similarity correlates with disease severity

**Figure 6:**
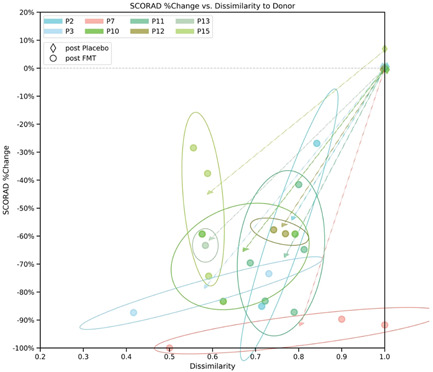
Correlation between clinical improvement and similarity in bacterial strains of patients and donors following FMT. Dots correspond to individual patient samples, plotted by their dissimilarity to their donor (x‐axis) versus the reduction of the SCORAD score from baseline (Week 4) at the time of the sample (y‐axis). Arrows demonstrate the path from placebo samples (diamond shape markers) to post‐FMT samples. Ellipse encompass patient post‐FMT samples. FMT, fecal microbiota transplantation

Finally, we found a significant correlation between the improvement in disease severity (change in the SCORAD score) and the sample dissimilarity between donor and their patients (*R* = .68, *p* < .001), (Figure 6). These results demonstrate an association between the degree of strain transmission from donor to patient and clinical improvements, suggestive of a possible casual effect of bacterial strain transmission from donor to patient in treatment of AD.

## 4 DISCUSSION

In this study we examined the effect of FMT treatment on moderate to severe AD. Four FMT sessions resulted in significant improvement in signs and symptoms as compared to baseline. Despite our small cohort these results provide the first clinical evidence for the importance of the gut microbiota in AD patients and demonstrate that FMT may be an effective therapeutic intervention. There were no side effects to the treatment during the study and the follow up period.

We further found that FMTs resulted in strain transmission events to patients and that the higher the similarity was between the microbiome of donors and the patients after the FMT, the higher the clinical improvement was. Some strains were transmitted to multiple patients, with SGB_1626—Prevotella copri being a notable case as it was transmitted to 7 of the 8 eligible patients for whom stool samples were available. These results suggest that the gut microbiota may have a causal effect in AD and may be a viable therapeutic target for AD.

There was a variability in the response to the FMT treatment among the patients. Some reacted immediately after the first FMT while others improved substantially only few weeks following the fourth FMT.

Five patients retained the improvement during the follow up period. Four patients relapsed. Two had an early relapse, on Weeks 10 and 12. The other 2 patients, experienced late relapse and responded well to a fifth FMT, revealing that microbiota change after the FMT can be temporary.

Two patients had a substantial improvement after placebo transplantation, not associated with microbial composition alteration, reflecting the placebo effect or natural course of the disease.

This study demonstrates for the first time, that FMT can be an effective and safe procedure for moderate to severe AD patients. The results of the dupilumab studies, demonstrated a mean change of the SCORAD score at Week 16 of 57%–62% from baseline,^18^, ^19^, ^20^ compared to 59.2% reduction 2 weeks following the last FMT in the present study.

The association between microbial transfer and clinical outcome was most strongly observed in two patients who responded to the FMT, experienced a relapse, and regained response after additional FMT, suggesting FMT can be therapeutically used for the induction of initial disease control but also maintenance of disease control of AD. Although this is a pilot study, its unique design enabled us to demonstrate that these changes were not apparent in the control arm. Nevertheless, this study has limitations, including the small number of participants, the lack of double blinded design and lack of information regarding additional factors that may impact the gut microbial composition, such as diet.

FMTs have been shown to be the most effective therapy for recurrent Clostridium difficile infection.^21^ Although FMTs are currently tested as therapeutic interventions for multiple disease conditions, to our knowledge, no other indication have shown such a robust clinical result.

The skin and the intestine both have an important role as immunological barriers and immune regulation. Intestinal microbiota influence the skin through modulatory effect of the gut on systemic immunity.^22^ Increasing evidence support the existence of a gut‐skin axis. Studies linked inflammatory skin diseases such as AD, psoriasis, rosacea, and acne vulgaris, to an imbalanced gut microbiome. Therefore, a change of the gut microbiota might improve those skin conditions.^6^

Probiotic‐fed mice exhibited increased dermal thickness, improved folliculogenesis, and increased sebocyte production resulting in thicker and shinier fur.^23^ Other studies failed to show any beneficiary effect of probiotic treatment on the severity of AD despite an alteration in the gut microbial composition.^10^ Recently published study evaluated the effects of six Bifidobacteria adolescentis strains on skin lesions, gut microbial profiles, and their immunomodulatory properties of 2,4‐dinitrofluorobenzene (DNFB) induce AD symptoms mice. Treatment with Bifidobacteria adolescentis alleviated the AD‐like clinical symptoms, decreased serum IgE levels, suppressed IL‐4, IL‐5, IL‐13, and CCL22 levels, and increased interferon‐γ and IL‐10 levels, thus promoting Th1‐type and suppressing Th2‐type immune responses.^24^ A recent study using mouse model of AD showed that FMT was associated with restoration of gut microbiota and immunological balance (Th1/Th2) along with suppression allergic response.^11^

In humans, local application of Roseomonas mucosa was effective for AD.^25^ Oral Probiotic supplementation reduced in several studies the incidence of skin manifestations of inflammatory bowel diseases, probably by excreting anti‐inflammatory effect, improving the barrier mechanism^26^, ^27^ decreasing transepidermal water loss, increasing skin hydration, thus decreasing the risk of AD in children^11^, ^23^, ^28^, ^29^, ^30^ and alleviating its manifestations.^31^ A change in the gut microbiome caused by antibiotic treatment, prenatal or during the first 2 years of life, as well as C‐section, increase the risk of atopic march and eczema in children.^32^, ^33^, ^34^ High level of certain intestinal bacteria as well as low bacterial diversity increase the risk of AD^35^, ^36^, ^37^ Furthermore, elevated calprotectin levels, were found to correlate with AD severity.^38^, ^39^

Further studies, randomized double blind placebo controlled, should be performed to confirm the clinical efficacy and determine the best regimen of FMT for AD. Additional multiomics analysis should be performed to provide insight towards the exact bacteria, metabolites and immune mediators involved.

## FUNDING INFORMATION

Funding information is not available.

## CONFLICT OF INTERESTS

The authors declare that there are no conflict of interests.

## AUTHOR CONTRIBUTIONS

*Conceptualization*: Jacob Mashiah, Nitsan Maharshak, Eran Segal; *Data Curation*: Anastasia Godneva, Tal Karady; *Formal analysis*: Tal Karady; *Investigation*: Nitsan Maharshak, Jacob Mashiah, Naomi Fliss‐Isakov, Dan Slodownik, OA, Liat Samuelov, Eran Ellenbogen, DH; *Methodology*: Jacob Mashiah, Nitsan Maharshak, Eran Segal; *Project administration*: Jacob Mashiah, Nitsan Maharshak, Eran Segal, ESp, Naomi Fliss‐Isakov; *Resources*: Eran Segal, Nitsan Maharshak, ESp; *Supervision*: Jacob Mashiah, Nitsan Maharshak, Eran Segal; *Visualization*: Tal Karady; *Writing—original draft*: Jacob Mashiah, Tal Karady, Nitsan Maharshak, Eran Segal; *Writing—review and editing*: Jacob Mashiah, Tal Karady, Nitsan Maharshak, Eran Segal, Naomi Fliss‐Isakov.
